# Changes in Children’s Body Composition and Posture during Puberty Growth

**DOI:** 10.3390/children8040288

**Published:** 2021-04-08

**Authors:** Wojciech Rusek, Joanna Baran, Justyna Leszczak, Marzena Adamczyk, Rafał Baran, Aneta Weres, Grzegorz Inglot, Ewelina Czenczek-Lewandowska, Teresa Pop

**Affiliations:** 1 Rehabilitation Centre Rehamed-Center Sp z o.o. Tajecina 66A, 36-002 Tajęcina, Poland; rusekaw@interia.pl (W.R.); m.adamczyk@rehamed-center.pl (M.A.); 2 Institute of Health Sciences, Medical College, University of Rzeszów, Al. mjr. W. Kopisto 2a, 35-310 Rzeszów, Poland; leszczakjustyna.ur@gmail.com (J.L.); anetaweres.ur@gmail.com (A.W.); e.czenczek@univ.rzeszow.pl (E.C.-L.); popter@interia.pl (T.P.); 3 Natural and Medical Center for Innovative Research, ul. Litawora 2, 35-310 Rzeszów, Poland; 4 RehaKlinika Sp. z o.o., ul. Pogodne Wzgórze 12, 35-317 Rzeszów, Poland; 5 Solution-Statistical Analysis, ul. Stojałowskiego 4/73, 35-120 Rzeszów, Poland; rafal.barann@gmail.com; 6 Institute of Medical Sciences, Medical College, University of Rzeszów, Al. mjr. W. Kopisto 2a, 35-310 Rzeszów, Poland; iglooo@interia.pl; 7 Individual Medical Practice Grzegorz Inglot, ul. Myśliwska 9, 36-060 Glogow Malopolski, Poland

**Keywords:** asymmetry, body composition, body posture, puberty

## Abstract

The main goal of our study was to determine how the age of children, puberty and anthropometric parameters affect the formation of body composition and faulty body posture development in children. The secondary goal was to determine in which body segments abnormalities most often occur and how gender differentiates the occurrence of adverse changes in children’s body posture and body composition during puberty. The study group consisted of 464 schoolchildren aged from 6–16. Body posture was assessed with the Zebris system. The composition of the body mass was tested with Tanita MC 780 MA body mass analyzer and the body height was measured using a portable stadiometer PORTSTAND 210. The participants were further divided due to the age of puberty. Tanner division was adopted. The cut-off age for girls is ≥10 years and for boys it is ≥12 years. The analyses applied descriptive statistics, the Pearson correlation, stepwise regression analysis and the t-test. The accepted level of significance was *p* < 0.05. The pelvic obliquity was lower in older children (beta = −0.15). We also see that age played a significant role in the difference in the height of the right pelvis (beta = −0.28), and the difference in the height of the right shoulder (beta = 0.23). Regression analysis showed that the content of adipose tissue (FAT%) increased with body mass index (BMI) and decreased with increasing weight, age, and height. Moreover, the FAT% was lower in boys than in girls (beta negative equal to −0.39). It turned out that older children (puberty), had greater asymmetry in the right shoulder blade (*p* < 0.001) and right shoulder (*p* = 0.003). On the other hand, younger children (who were still before puberty) had greater anomalies in the left trunk inclination (*p* = 0.048) as well as in the pelvic obliquity (*p* = 0.008). Girls in puberty were characterized by greater asymmetry on the right side, including the shoulders (*p* = 0.001), the scapula (*p* = 0.001) and the pelvis (*p* < 0.001). In boys, the problem related only to the asymmetry of the shoulder blades (*p* < 0.001). Girls were characterized by a greater increase in adipose tissue and boys by muscle tissue. Significant differences also appeared in the body posture of the examined children. Greater asymmetry within scapulas and shoulders were seen in children during puberty. Therefore, a growing child should be closely monitored to protect them from the adverse consequences of poor posture or excessive accumulation of adipose tissue in the body.

## 1. Introduction

Postural defects as well as excessive weight and obesity are among the most common issues faced by children in developmental age [1]. It is a well-known fact that incorrect positioning in the womb, asymmetry of muscle tone, premature birth [2] and adoption of incorrect posture while sitting or playing are just some of the factors contributing to the development of faulty body posture in children. This is true regardless of the issue: knock knees, flat feet, scoliosis, etc. The problem at hand affects the entire body of a child, because incorrect alignment of structures in one area brings about more misalignment in another. According to current data, 34–50% children and adolescents have different degrees of incorrect posture [3,4]. Professional literature specifies two critical periods for postural development: the first one around the age of 7 is referred to as “school years”, and the second as “puberty” [5]. Incorrect body posture in childhood has consequences in adulthood, including reduction of circulatory and respiratory efficiency and vital lung capacity, pain in the spine and related structures, as well as displacement of internal organs [6,7,8].

The situation is similar with the composition of body mass, in particular with the content of adipose tissue and the development of excessive weight and obesity in children [9]. The origins of the issue may be traced back to fetal age, when the following factors contributing to the development of obesity in later age are pointed out: excessive gestational weight gain (GWG) [10,11,12], poorly balanced diet [13], and smoking [14]. All these factors may contribute to the abnormal birth weight of a child and excessive body mass index (BMI) in the subsequent years of life. Another study suggests that moderate to vigorous physical activity (MVPA) and vigorous physical activity (VPA) may attenuate the increased risk of an unfavorable body composition and BMI due to high maternal pre-pregnancy BMI and rapid infant weight gain in boys, but not in girls [15].

Fat content changes in the human body with age and is also gender-specific. Girls and women have a higher body fat content than boys and men. This has to do with the reproductive and endocrine function of the body. Moreover, the distribution of adipose tissue also varies with gender and age [16]. During puberty, dramatic hormonal fluctuations as well as a rapid growth in body size occur and are accompanied by marked changes in body composition [17]. The main goal of our study was to determine how the age of children, puberty and anthropometric parameters affect the formation of body composition and faulty body posture development in children. The secondary goal was to determine in which body segments abnormalities most often occur and how gender differentiates the occurrence of adverse changes in children’s body posture and body composition during puberty.

## 2. Materials and Methods

### 2.1. Participants

Before the beginning of the study sample size was calculated with reference to the total number of children (*n* = 3790) living in the Trzebownisko Municipality, a rural area in south-eastern Poland. We used a 95% confidence level and a confidence interval (CI) of 0.05. It was calculated that the minimum sample size should be 349. The study took into account 464 school-age children, from 6 to 16 years of age (234 boys and 230 girls). 464 schoolchildren aged from 6–16 participated in the research, 234 of whom were boys and 230 of girls. This study was conducted in eight randomly selected schools (five primary and three secondary schools) in Trzebownisko commune. To ensure the reliability of the measurements, all students were tested in the morning on an empty stomach by the same members of a qualified team. The following inclusion criteria were used in the study: age from 6 to 16 years, consent of parents and children to the study, no certificate of intellectual or motor disability, students in fasting state. Exclusion criteria: metal implants, electronic implants, menstruation, a certificate of mental or motor disability, epilepsy, and failure to refrain from eating on the morning of the examination. Children with neonatal hypertonia or muscle hypotension, chronic neurological diseases or previous injuries and surgery in the last 6 months prior to the study were excluded from the study group.

The participants were informed about the course of the study. The tests were conducted after written consent was received from the headmasters of the schools, parents of the children participating in the project and the children themselves.

The participants were further divided according to the age of puberty. Tanner division was adopted. The cut-off age for girls is ≥10 years and for boys it is ≥12 years [18].

### 2.2. Body Posture

Body posture was assessed with the Zebris system. The Zebris system is a very extensive diagnostic system with a wide range of applications. It includes accessories for assessing body posture, gait, range of motion, balance and ground forces reaction. It is used in medicine, dentistry and sports, etc. This system shows high sensitivity, precision and measurement reliability [19,20]. Measurement error of the system is estimated at about 5.5% [21]. The Zebris system consists of a measuring unit, a system of micro transmitters and an ultrasonic pointer with which topographic points from the skeleton are scanned, and then compiled in a computer system in the form of a report containing numerical data specifying lengths, angles and degrees for individual parameters, as well as graphs and figures of spine lines and other anthropometric parameters.

Based on the defined topographic points, the software computes the values of selected body posture parameters [22,23,24]. The following parameters were taken into account in the assessment of posture: total length of spine (mm), thoracic length (mm), lumbar length (mm), pelvic torsion (degree), pelvic obliquity (degree), pelvic/shoulder obliquity (degree), scapula distance right (mm), scapula distance left (mm), scapula distance difference (mm), pelvic height difference right (mm), pelvic height difference left (mm), shoulder height difference right (mm), shoulder height difference left (mm), lateral inclination left (degree), lateral inclination right (degree). For parameters such as the shoulders or pelvis, the right or left side means the upper arm. In the case of lateral inclination, the right or left side indicates to which side the torso is inclined.

For a detailed comparison of children’s posture parameters and body weight composition by gender, as well as relation between body composition and body posture parameters, please see another publication from our project [25].

### 2.3. Anthropometric Measurements and Bioelectrical Impedance

The composition of the body mass was tested with Tanita MC 780 MA (Tanita, Tokyo, Japan) body mass analyzer, which operates on the basis of the phenomenon of bioelectrical impedance (BIA). The bioelectrical impedance method is recognized in the medical and scientific world. Many scientists and practitioners use it. Numerous studies have been carried out on the accuracy and reliability of measurements in various age and clinical groups [26,27,28,29,30,31]. By analyzing the composition of body weight, the content of adipose tissue (FAT% and kg), lean tissue (fat free mass (FFM)kg), muscle tissue (PMM kg), total water content (TBW%), basic metabolism (BMR) and impedance (IMP) were assessed. Additionally, the parameters of the body mass component analysis segments were measured in the trunk (TR), lower right and left limb (RLP, LLL), and upper right and left limb (RAP, LAL).

A PORTSTAND 210 (Charder, Taichung, Taiwan) portable stadiometer was used to measure the height of the subjects. An accuracy level of 0.1 cm was adopted. Body weight was measured with an accuracy of 0.1 kg using a Tanita analyzer.

With data on weight and height, the child’s BMI was calculated.

### 2.4. Statistical Analysis

The analyses applied descriptive statistics (mean, median, number of subjects, first quartile, third quartile, standard deviation). Statistical analysis was performed on the Statistica 10.0 software developed by StatSoft (Tulsa, OK, USA), and Microsoft Excel. In order to check the correlation between the age and the parameters of posture and body weight, the Pearson correlation was used. Then, the analysis was extended to assess the impact of selected factors on changes in the parameters of body posture and body weight using stepwise regression analysis. The final stage was to evaluate the differences in posture parameters and body weight composition between pre-puberty and during puberty. These differences were assessed by the t-test. The accepted level of significance was *p* < 0.05.

### 2.5. Ethical Approval

The approval of the Bioethics Committee of the University of Rzeszów No. 2016/06/28 on 28 June 2016 was obtained for conduct of the research.

## 3. Results

The study group of 464 children in total included a similar number of girls (230) and boys (234). The mean age of the examined girls and boys (11.55 vs. 11.49) and BMI (18.64 vs. 18.94) were also similar (Table 1).

First, a check was made as to whether the age of the children correlated with the parameters of posture and body weight. Using Pearson’s correlation, several statistically significant correlations were obtained. Older children were characterized by a greater total length of the spine, longer individual sections of the spine (a natural feature), but greater asymmetry in the right scapula, right shoulder and right side of the pelvis.

In the case of body mass composition. total fat decreased with age. Taking into account individual body segments, we can see that limb fatness decreases with age, while it increases in the trunk.

Taking into account the sex of the respondents, it turned out that older girls had more body fat, older boys had more muscle, and both older girls and boys had more water.

In the case of body posture parameters, most of the correlations by gender are consistent with the correlations in the whole group (Table 2).

Taking into account that the demonstrated correlations are not too strong, it was decided to check the influence of other factors, including anthropometric factors. For this purpose, step regressions were performed to assess the influence of factors on the parameters of posture and body weight. In the first case, individual body posture parameters were selected as the dependent variable, and the independent variables were: age, sex, height, weight, BMI, FAT%, FFM%, TBW%, PMM%. Thus, the influence of both anthropometric factors and body mass composition on body posture parameters was checked. These data are presented in Table 3 and Table S1.

The pelvic obliquity in relation to the ground was lower in older children (beta = −0.15). We also see that age played a significant role in the difference in the height of the right pelvis (beta = −0.28), and the difference in the height of the right shoulder (beta = 0.23). The notation “no variables in the model” means that none of the independent variables significantly influenced the given parameter of body posture and the model could not be built.

In the second regression analysis, body composition parameters were selected as dependent variables, and age, sex, height, weight and BMI were selected as independent variables. These data are presented in Table 4.

Regression analysis showed that FAT% increased with BMI and decreased with increasing weight, age, and height. Moreover, the FAT% was lower in boys than in girls (beta negative equal to −0.39). Taking into account the total body water, it increased with the age of the respondents and was higher in boys. Importantly, water content significantly decreased with the BMI of the respondents (beta = −1.35). Constant values were immediately removed from the table below.

As a complement to the analyzes, it was checked whether the puberty period differentiates the parameters of posture and body mass composition. It turned out that older children (puberty), in addition to greater spine length parameters (which is natural), also had greater asymmetry in the right shoulder blade (*p* < 0.001) and right shoulder (*p* = 0.003). On the other hand, younger children (who were still before puberty) had greater anomalies in the left trunk inclination (*p* = 0.048) as well as in the pelvic obliquity (*p* = 0.008). Additionally, it has been noticed that during adolescence, the development of adipose tissue is dominant in girls, and that in boys there is higher total body water and lean tissue content.

It was also noticed that younger children had lower body fat (*p* = 0.019) and an automatically higher content of total body water (*p* = 0.015). Older children had greater body fat (*p* < 0.001).

Girls in puberty were characterized by greater asymmetry on the right side, including the shoulders (*p* = 0.001), the scapula (*p* = 0.001) and the pelvis (*p* < 0.001). In boys, the problem related only to the asymmetry of the shoulder blades (*p* < 0.001). Interestingly, when comparing girls and boys before puberty, boys show greater asymmetries in body posture. These data are presented in Table 5. Detailed data presenting descriptive statistics for the following results are presented in additional materials (Tables S2–S6).

## 4. Discussion

The studies performed form a part of a major project which shows dependencies between the parameters describing body posture and age, puberty and anthropometric parameters in children from rural areas [25,26]. The project is important for practical purposes, as well as for the prevention of postural defects and excessive weight in children and adolescents. There are many studies in the literature assessing body posture, but there are relatively few that take into account factors such as body composition, age and sex. More importantly, in literature there are no scientific publications assessing the components of body weight, position of shoulder blades and the pelvis in puberty in children from rural areas. Accordingly, our research project is one of the first studies to evaluate these parameters.

The authors’ own research has shown that asymmetry on the right side increased with age. This concerns the shoulders, pelvis and the deviation of the scapula from the frontal plane. A similar tendency was shown by Yang et al. in their research. They showed that the asymmetry in the area of the shoulder blades and shoulders more often affects older children (>15 years of age) [32]. Pelvic asymmetry in children was confirmed by Drnach et al. [33]. The study of Kapo et al. showed that there is a negative trend of increasing body mass index within the first and youngest age group. The trend of increasing deformity of the shoulder belt has been noted, often inclining towards the formation of milder forms of kyphotic posture. Other forms of deformity brought to light in the survey results are the negative trend of increasing pelvic rotation as well as pelvis rotation which inclines towards the formation of lordotic posture for all three age groups [34].

Considering the components of body weight, our own results showed a reduction in body fat and an increase in water content. Research by Wilczyński et al. and Leskinen et al. show a tendency for the percentage of adipose tissue to decrease with age and for increased water content in both girls and boys [35,36].

Wyszyńska et al. indicate no differences in body posture between girls and boys. In our study, gender only played a role in the right scapula position, where boys had higher scores [37,38]. Rusek et al. showed that sex differentiates the position of the pelvis and the occurrence of asymmetry within it. In addition, adipose tissue content affects the asymmetry of the scapulae and pelvis in the frontal plane, which is also affected by the muscle tissue. [25]. Children with the lowest content of muscle tissue showed the highest difference in the height of the inferior angles of the scapulas in the coronal plane [37]. This is consistent with the results of our own research, where lower muscle tissue content was associated with greater asymmetry of the shoulder blades.

There were significant differences concerning body composition indices containing soft lean mass (SLM) and lean body mass (LBM) in female adolescents with postural deformities in comparison with normal posture. These indices (LBM and SLM) are protective from postural deformities in female normal weight adolescents [39]. In our own study, only one negative relationship was found between lean body mass and the position of the left scapula.

The regression analysis of the body mass composition of children in relation to their sex, age, BMI, height and body weight showed the influence of all these factors on the parameters of body mass composition. However, the most interesting seem to be taking into account the results of puberty. Loncar-Dusek and Pećina report similar findings in their research, which shows more frequent occurrence of scoliosis in adolescence in relation to the period before puberty [40]. Similarly, the research of Demirbüken el al. proves, that body posture is related to age and weight in early adolescence. Adolescence is an important period for identification of postural disorders and one in which it is possible to take precautions for the later ages [41]. Body composition during puberty is a marker of metabolic changes that occur during this period of growth and maturation, and, thus, holds key information regarding current and future health. During puberty, the main components of body composition (total body fat, lean body mass, bone mineral content) all increase; however, considerable sexual dimorphism exists [42]. Both sexes experience rapid increases in total body fat, although the proportion of body fat increases more slowly in boys as a result of a simultaneous rapid increase in fat-free mass [43]. In our study, we found differences in body weight composition between boys and girls across all parameters. The girls were characterized by a higher content of adipose tissue in both general and segmental terms. The boys, on the other hand, had a higher content of lean tissue, muscle tissue, and total body water content.

The research focused on changes in children’s body composition and posture during puberty. Changes in body posture in adolescence are a topic often described by various authors, but studies in this field should still be carried out because, as our research shows, there are still many factors influencing body posture that have not been studied.

### Strengths and Limitations of the Study

The research was performed only among children from rural areas, which can also be seen as a positive factor, because such specialized research is rarely performed in this study group.

In addition, we cannot exclude the influence of confounding factors on changes in the composition of body weight or body posture (e.g., type of diet, genetic background, etc.).

The influence of physical activity on posture and body weight composition has not been investigated either, therefore it should be taken into account when planning further research.

Among the strengths of the study is undoubtedly the homogeneity and size of the study group as well as the use of objective measuring tools to assess posture and body weight composition.

## 5. Conclusions

The research conducted has shown that the period of puberty is a time of significant changes in the body of a child. These changes vary depending on the sex of the respondents. Significant differences appeared in the body posture and body composition of the examined children. Greater asymmetry within scapula and shoulders were seen in children during puberty. Girls were characterized by a greater increase in adipose tissue and boys by muscle tissue. Older children (both girls and boys) were characterized by a higher water content in the body. Therefore, a growing child should be closely monitored to protect them from the adverse consequences of poor posture or excessive accumulation of adipose tissue in the body.

## Figures and Tables

**Table 1:** Characteristics of the study group.

Variables	*n*	Mean (SD)	Q_1_	Median	Q_3_
**All**						
Age (years)	464	11.52 (2.99)	9.00	12.00	14.00
Height (cm)	464	152.48 (17.77)	137.00	155.50	167.00
Weight (kg)	464	45.39 (16.81)	30.40	44.75	56.45
Body mass index (BMIkg/m^2^)	464	18.80 (3.75)	16.10	18.24	20.97
Total length of spine (mm)	464	430.38 (52.62)	388.50	433.00	469.50
Thoracic Length (mm)	464	307.89 (35.87)	281.00	307.00	332.00
Lumbar Length (mm)	464	90.10 (20.04)	76.00	91.00	105.00
Pelvic torsion (degree)	464	3.66 (3.43)	1.30	2.90	4.80
Pelvic obliquity (degree)	464	2.11 (1.77)	0.70	1.70	2.90
Pelvic/shoulder obliquity (degree)	464	2.97 (7.83)	1.10	2.15	3.70
Scapula distance right (mm)	464	46.21(14.33	37.00	46.00	56.00
Scapula distance left (mm)	464	47.06 (32.82)	37.00	45.00	54.00
Scapula distance difference (mm)	464	7.06 (6.15)	3.00	5.00	9.50
Pelvic height difference right (mm)	238	4.41(6.64)	0.00	0.10	7.30
Pelvic height difference left (mm)	226	4.08 (6.44)	0.00	0.00	6.10
Shoulder height difference right (mm)	155	6.48 (5.24)	2.70	5.40	8.60
Shoulder height difference left (mm)	309	11.59 (8.47)	4.80	10.10	16.40
Lateral inclination right (degree)	198	0.54 (0.88)	0.00	0.00	0.85
Lateral inclination left (degree)	266	0.78 (1.07)	0.00	0.20	1.40
The content of adipose tissue in % (Fat %)	464	21.61 (6.10)	16.80	20.40	25.40
The content of adipose tissue in kg (Fat kg)	464	10.25 (6.09)	5.65	8.65	13.25
Lean tissue in kg (FFM kg)	464	35.11(12.01)	24.35	34.80	43.50
Total water content in % (TBW %)	464	25.70 (8.79)	17.85	25.50	31.85
Muscle tissue in kg (PMM kg)	464	33.29 (11.44)	22.95	33.00	41.30
**Girls**						
Age (years)	230	11.55 (2.96)	9.00	12	14.00
Height (cm)	230	150.46 (15.69)	136.00	155	163.00
Weight (kg)	230	43.63 (15.38)	29.70	44.35	54.60
BMI (kg/m^2^)	230	18.64 (3.87)	15.90	18.00	20.90
Total length of spine (mm)	230	426.77 (46.97)	391.00	433.50	461.00
Thoracic Length (mm)	230	304.83 (33.52)	280.00	306.00	328.00
Lumbar Length (mm)	230	89.57 (18.79)	77.00	90.00	104.00
Pelvic torsion (degree)	230	3.72 (3.36)	1.40	2.90	4.80
Pelvic obliquity (degree)	230	2.04 (1.83)	0.70	1.60	2.80
Pelvic/shoulder obliquity (degree)	230	2.38 (1.85)	1.00	1.90	3.40
Scapula distance right (mm)	230	42.94 (12.85)	34.00	44.00	52.00
Scapula distance left (mm)	230	45.56 (44.56)	34.00	43.00	51.00
Scapula distance difference (mm)	230	6.94 (5.83)	3.00	5.00	10.00
Pelvic height difference right(mm)	112	3.85 (6.24)	0.00	0.00	5.80
Pelvic height difference left(mm)	118	4.17 (6.53)	0.00	0.20	6.10
Shoulder height difference right (mm)	83	6.24 (5.50)	2.50	4.80	8.40
Shoulder height difference left (mm)	147	11.83 (8.04)	5.30	10.25	17.30
Lateral inclination right (degree)	98	0.51 (0.85)	0.00	0.00	0.80
Lateral inclination left (degree)	132	0.73 (1.00)	0.00	0.20	1.40
The content of adipose tissue in % (Fat %)	230	23.96 (5.52)	19.50	23.40	27.20
The content of adipose tissue in kg (Fat kg)	230	11.07 (6.24)	6.20	9.55	14.40
Lean tissue in kg (FFM kg)	230	32.56 (9.76)	22.90	34.00	39.80
Total water content in % (TBW %)	230	23.83 (7.14)	16.80	24.90	29.10
Muscle tissue in kg (PMM kg)	230	30.88 (9.28)	21.70	32.25	37.80
**Boys**						
Age (years)	234	11.49 (3.02)	9.00	12	14.00
Height (cm)	234	154.46 (19.43)	138.00	156.50	172.00
Weight (kg)	234	47.13 (17.97)	31.80	45	60.00
BMI (kg/m^2^)	234	18.94 (3.63)	16.40	18.56	21.05
Total length of spine (mm)	234	433.93 (57.52)	385.00	433.00	483.00
Thoracic Length (mm)	234	310.89 (37.87)	281.00	307.00	337.00
Lumbar Length (mm)	234	90.63 (21.23)	74.00	91.50	106.00
Pelvic torsion (degree)	234	3.59 (3.50)	1.30	2.85	4.80
Pelvic obliquity (degree)	234	2.18 (1.71)	0.80	1.95	3.00
Pelvic/shoulder obliquity (degree)	234	3.55 (10.86)	1.20	2.50	4.10
Scapula distance right (mm)	234	49.42 (14.99)	39.00	49.00	60.00
Scapula distance left (mm)	234	48.54 (13.57)	40.00	48.00	59.00
Scapula distance difference (mm)	234	7.18 (6.46)	3.00	6.00	9.00
Pelvic height difference right(mm)	126	4.95 (6.98)	0.00	0.40	9.00
Pelvic height difference left (mm)	108	3.98 (6.37)	0.00	0.00	6.10
Shoulder height difference right (mm)	72	6.79 (4.93)	3.10	6.60	9.40
Shoulder height difference left (mm)	162	11.38 (8.84)	4.50	9.40	16.20
Lateral inclination right (degree)	100	0.57 (0.90)	0.00	0.00	0.90
Lateral inclination left (degree)	134	0.83 (1.13)	0.00	0.20	1.40
The content of adipose tissue in % (Fat %)	234	19.30 (5.77)	15.40	17.60	21.70
The content of adipose tissue in kg (Fat kg)	234	9.44 (5.85)	5.30	8.20	11.20
Lean tissue in kg (FFM kg)	234	37.62 (13.43)	25.90	36.35	49.30
Total water content in % (TBW %)	234	27.54 (9.83)	19.00	26.60	36.10
Muscle tissue in kg (PMM kg)	234	35.65 (12.82)	24.50	34.45	46.80

M: mean; Me: median; n: number of subjects; Q_1_: first quartile; Q_3_: third quartile SD: standard deviation.

**Table 2:** Correlation between age and body posture and body composition parameters.

Age &	All		Girls		Boys	
**Body Posture**	**R**	***p***	**R**	***p***	**R**	***p***
Total length (mm)	0.839	<0.001	0.823	<0.001	0.864	<0.001
Thoracic Length (mm)	0.696	<0.001	0.665	<0.001	0.730	<0.001
Lumbar Length (mm)	0.718	<0.001	0.664	<0.001	0.767	<0.001
Pelvic torsion (degree)	−0.072	0.122	−0.181	0.006	0.028	0.669
Pelvic obliquity (degree)	−0.151	0.001	−0.164	0.013	−0.137	0.036
Pelvic/shoulder obliquity (degree)	0.046	0.319	0.005	0.941	0.066	0.314
Scapula distance RIGHT (mm)	0.343	<0.001	0.182	0.006	0.500	<0.001
Scapula distance LEFT (mm)	0.055	0.235	−0.059	0.376	0.451	<0.001
Scapula distance difference (mm)	−0.072	0.120	−0.055	0.402	−0.087	0.187
Pelvic height difference RIGHT (mm)	0.092	0.047	0.173	0.009	0.025	0.700
Pelvic height difference LEFT (mm)	−0.085	0.068	−0.171	0.009	0.000	0.998
Shoulder height difference RIGHT (mm)	0.230	0.005	0.270	0.013	0.176	0.154
Shoulder height difference LEFT (mm)	−0.024	0.667	−0.039	0.644	−0.014	0.856
Lateral inclination LEFT (degree)	−0.063	0.173	−0.083	0.213	−0.047	0.476
Lateral inclination RIGHT (degree)	0.005	0.912	0.043	0.517	−0.028	0.665
**Body Composition**						
The content of adipose tissue in % (Fat %)	−0.121	0.009	0.383	<0.001	−0.109	0.096
Lean tissue in % (FFM %)	0.126	0.007	−0.381	<0.001	0.092	0.160
Total water content in % (TBW %)	0.127	0.006	0.856	<0.001	0.881	<0.001
Muscle tissue in % (PMM %)	0.102	0.027	−0.372	<0.001	0.133	0.043
Right leg FAT %	−0.088	0.057	0.263	<0.001	−0.390	<0.001
Left leg FAT %	−0.107	0.021	0.233	<0.001	−0.388	<0.001
Right arm FAT %	−0.163	<0.001	0.064	0.333	−0.404	<0.001
Left arm FAT %	−0.027	0.560	0.291	<0.001	−0.342	<0.001
Total trunk FAT %	0.203	<0.001	0.359	<0.001	0.052	0.429

R—Pearson’s correlation ratio.

**Table 3:** Regression of factors influencing body posture parameters in children.

Model		Non-Standard Coefficient		Standardized Coefficient	*t*	*p*	95% CI (B)		R^2 -^Corrected
		B	SE	Beta					
Pelvic torsion (degree)	no variables in the model								
Pelvic obliquity (degree)	Age	−0.09	0.03	−0.15	−3.28	<0.001	−0.14	−0.04	0.021	
Pelvic/shoulder obliquity (degree)	no variables in the model								
Scapula distance RIGHT (mm)	Body height	0.34	0.03	0.42	10.33	0.001	0.28	0.41	0.245
	FAT %	−0.56	0.10	−0.24	−5.36	<0.001	−0.76	−0.35	
	Sex	2.52	1.27	0.09	1.98	0.048	0.02	5.01	
Scapula distance LEFT (mm)	PMM %	16.66	7.48	2.94	2.23	0.026	1.96	31.35	0.017
	FFM %	−15.12	7.03	−2.84	−2.15	0.032	−28.94	−1.30	
Scapula distance difference (mm)	PMM %	−0.12	0.05	−0.12	−2.51	0.013	−0.22	−0.03	0.011
Pelvic height difference RIGHT (mm)	Body height	0.16	0.04	0.42	3.99	<0.001	0.08	0.23	0.037
	Age	−0.63	0.23	−0.28	−2.71	0.007	−1.09	−0.17	
Pelvic height difference LEFT (mm)	Body height	−0.04	0.02	−0.10	−2.22	0.027	−0.07	0.00	0.008
Shoulder height difference RIGHT (mm)	Age	0.40	0.14	0.23	2.87	0.005	0.12	0.67	0.046
Shoulder height difference LEFT (mm)	FAT %	0.18	0.07	0.14	2.44	0.015	0.04	0.33	0.016
Lateral inclination LEFT (degree)	no variables in the model								
Lateral inclination RIGHT (degree)	no variables in the model								

95% confidence interval for the B index (Non-standard coefficient); B—non-standard coefficient; Beta—standard coefficient; R^2 -^—coefficient of determination; SE—standard error.

**Table 4:** Regression of factors influencing the parameters of body mass composition in children.

Model		Non-Standard Coefficient		Standardized Coefficient	*t*	*p*	95% CI (B)		R^2 -^Corrected
		B	SE	Beta					
FAT %	BMI	2.28	0.15	1.40	15.65	<0.001	1.99	2.57	0.810
	Sex	−4.73	0.26	−0.39	−18.33	<0.001	−5.24	−4.23	
	Body weight	−0.29	0.06	−0.80	−5.13	<0.001	−0.40	−0.18	
	Age	−0.46	0.10	−0.23	−4.65	<0.001	−0.66	−0.27	
	Body height	0.09	0.04	0.27	2.48	0.013	0.02	0.16	
FFM %	BMI	−2.22	0.15	−1.35	−14.48	<0.001	−2.52	−1.92	0.795
	Sex	4.70	0.27	0.38	17.34	<0.001	4.17	5.24	
	Body weight	0.26	0.06	0.71	4.40	<0.001	0.14	0.38	
	Age	0.48	0.10	0.23	4.54	<0.001	0.27	0.68	
	Body height	−0.08	0.04	−0.23	−2.07	0.039	−0.16	0.00	
TBW %	BMI	−1.62	0.11	−1.35	−14.48	<0.001	−1.84	−1.40	0.795
	Sex	3.44	0.20	0.38	17.34	<0.001	3.05	3.83	
	Body weight	0.19	0.04	0.71	4.40	<0.001	0.11	0.28	
	Age	0.35	0.08	0.23	4.52	<0.001	0.20	0.50	
	Body height	−0.06	0.03	−0.23	−2.06	0.040	−0.11	0.00	
PMM %	BMI	−1.87	0.08	−1.21	−22.76	<0.001	−2.03	−1.71	0.786
	Sex	0.15	0.03	0.45	5.90	<0.001	3.76	4.78	
	Body weight	4.27	0.26	0.37	16.41	<0.001	0.10	0.21	
	Age	0.35	0.09	0.18	4.16	<0.001	0.19	0.52	

95% confidence interval for the B index (Non-standard coefficient); B—non-standard coefficient; Beta—standard coefficient; R^2 -^—coefficient of determination; SE—standard error.

**Table 5:** The age of puberty and the parameters of posture and body weight in children.

Variables	Girls before vs. after Puberty *p*	Boys before vs. after Puberty *p*	Girls vs. Boys before Puberty *p*	Girls vs. Boys after Puberty *p*	All Children before vs. after Puberty *p*
**Body posture**					
Total length [mm]	<0.001	<0.001	0.008	<0.001	<0.001
Thoracic Length [mm]	<0.001	<0.001	0.008	<0.001	<0.001
Lumbar Length [mm]	<0.001	<0.001	0.241	<0.001	<0.001
Pelvic torsion	0.015	0.592	0.053	0.377	0.264
Pelvic obliquity	0.188	0.022	0.605	0.942	0.008
Pelvic/shoulder obliquity	0.690	0.403	0.139	0.139	0.617
Scapula distance RIGHT	0.001	<0.001	0.079	<0.001	<0.001
Scapula distance LEFT	0.492	<0.001	0.348	<0.001	0.470
Scapula distance difference	0.301	0.364	0.966	0.838	0.156
Pelvic height difference RIGHT	<0.001	0.950	<0.001	0.780	0.065
Pelvic height difference LEFT	0.039	0.901	0.136	0.588	0.136
Shoulder height difference RIGHT	0.001	0.251	0.033	0.995	0.003
Shoulder height difference LEFT	0.070	0.571	0.101	0.567	0.579
Lateral inclination LEFT	0.025	0.638	0.528	0.154	0.048
Lateral inclination RIGHT	0.008	0.644	0.016	0.594	0.288
**Body composition**					
The content of adipose tissue in % (Fat %)	0.092	0.063	0.016	<0.001	0.019
Lean tissue in % (FFM %)	0.234	0.103	0.015	<0.001	0.015
Total water content in % (TBW %)	0.726	0.105	0.015	<0.001	0.015
Muscle tissue in % (PMM %)	0.016	0.030	0.033	<0.001	0.054
Right leg FAT %	0.001	0.657	0.132	<0.001	0.246
Left leg FAT %	<0.001	<0.001	<0.001	<0.001	0.157
Right arm FAT %	<0.001	<0.001	<0.001	<0.001	0.005
Left arm FAT %	<0.001	<0.001	<0.001	<0.001	0.870
Total trunk FAT %	<0.001	<0.001	<0.001	<0.001	<0.001

*p*—*t*-test for independent groups (taking into account the correction resulting from the homogeneity of variance (homogeneity was calculated using Levene’s test).

## Data Availability

The data presented in this study is available upon request of the respective author. Due to the protection of personal data, the data is not publicly available.

## Supplementary Materials

The following are available online at https://www.mdpi.com/article/10.3390/children8040288/s1, Table S1: Regression of factors influencing body posture parameters in children. Table S2: Differences between girls and boys in body posture and body composition parameters before puberty, Table S3: Differences between girls and boys in body posture and body composition parameters after puberty. Table S4: Differences in girl’s body posture and body composition parameters before and after puberty. Table S5: Differences in boy’s body posture and body composition parameters before and after puberty. Table S6: Differences in body posture and body composition parameters before and after puberty in all children.
