# Supplementary material for: Changes in Children’s Body Composition and Posture during Puberty Growth
Source: Children (Basel). 2021 Apr 8;8(4):288. doi: 10.3390/children8040288 (PMC8068155; doi:10.3390/children8040288)
Supplement: Supplementary file 1 [file children-08-00288-s001.pdf]

Supplementary Table 1. Regression of factors influencing body posture parameters in children.

Model		Non-standard coefficient		Standarized coefficient	t	*p*	95% CI (B)		R^2-corrected
		B	SE	Beta					
Total length (mm)	Body height	2.39	0.11	0.81	21.76	<0.001	2.18	2.61	0.853
	Body weight	0.42	0.12	0.13	3.62	<0.001	0.19	0.65	
	Sex	-3.89	1.88	-0.04	-2.06	0.040	-7.59	-0.18	
Thoracic Length (mm)	Body height	1.48	0.16	0.73	9.19	<0.001	1.16	1.80	0.649
	Body weight	0.45	0.12	0.21	3.64	<0.001	0.20	0.69	
	Age	-1.57	0.76	-0.13	-2.07	0.039	-3.06	-0.08	
Lumbar Length (mm)	Body height	0.54	0.08	0.48	6.74	<0.001	0.38	0.70	0.557
	Age	1.92	0.48	0.29	4.04	<0.001	0.99	2.86	

It can be seen that the length of the spine depended on height (p <0.001), weight (p < 0.001) and sex (p = 0.039). Children who were taller (beta = 0.81), heavier (beta = 0.13) and girls (beta = - 0.04) had a longer spine. The second indicator of body posture was the length of the thoracic segment. This length significantly depended on the height, weight and age of the participants. Children who were taller (beta = 0.73), heavier (beta = 0.21) and to a lesser extent, younger children (beta = -0.13) had higher values of the thoracic segment length. The length of the lumbar section increased significantly with the growth (beta = 0.48) and with the age of children (beta = 0.29).

Supplementary Table 2. Differences between girls and boys in body posture and body composition parameters before puberty.

Variables before puberty	Girls						Boys						*p*
	×	SD	Me	Min.	Max.	*N*	×	SD	Me	Min.	Maks.	*N*	
**Body posture**													
Total length [mm]	374.27	28.88	371.00	314.00	457.00	71	387.04	32.96	385.00	308.00	486.00	112	0.008
Thoracic Length [mm]	273.59	23.23	272.00	176.00	332.00	71	283.65	25.38	282.50	217.00	392.00	112	0.008
Lumbar Length [mm]	73.28	15.59	74.00	34.00	105.00	71	76.11	15.99	74.00	23.00	110.00	112	0.241
Pelvic torsion [degree]	4.53	3.77	3.50	0.40	17.90	71	3.47	3.47	2.65	0.00	29.40	112	0.053
Pelvic obliquity [degree]	2.30	2.12	1.70	0.10	8.10	71	2.45	1.81	2.30	0.00	7.70	112	0.605
Pelvic/shoulder obliquity [degree]	2.45	1.90	2.00	0.00	8.90	71	2.93	2.21	2.60	0.00	10.40	112	0.139
Scapula distance RIGHT [mm]	38.83	12.92	40.00	10.00	69.00	71	42.32	13.10	41.50	11.00	84.00	112	0.079
Scapula distance LEFT [mm]	50.01	78.11	40.00	14.00	688.00	71	42.96	11.77	42.00	15.00	78.00	112	0.348
Scapula distance difference [mm]	7.54	6.73	6.00	0.00	32.00	71	7.58	7.01	6.00	0.00	41.00	112	0.966
Pelvic height difference RIGHT [mm]	1.87	3.18	0.00	0.00	12.10	71	4.92	6.36	0.30	0.00	23.20	112	<0.001
Pelvic height difference LEFT [mm]	5.61	7.33	2.20	0.00	24.10	71	4.04	6.65	0.00	0.00	32.40	112	0.136
Shoulder height difference RIGHT [mm]	3.94	3.23	2.90	0.20	14.90	29	6.08	4.34	5.40	0.10	19.30	33	0.033
Shoulder height difference LEFT [mm]	13.72	8.37	14.00	0.70	32.80	42	10.97	8.87	8.40	0.10	39.90	79	0.101
Lateral inclination LEFT [degree]	0.98	1.16	0.50	0.00	4.90	71	0.86	1.20	0.10	0.00	6.20	112	0.528
Lateral inclination RIGHT [degree]	0.31	0.66	0.00	0.00	2.90	71	0.60	0.92	0.00	0.00	4.40	112	0.016
**Body composition**													
Right Leg FAT [%]	29.99	5.55	29.60	8.50	48.30	71	26.23	5.99	25.40	5.90	47.80	112	0.132
Left Leg FAT [%]	30.64	4.90	29.80	23.80	50.30	71	26.71	6.05	25.95	6.40	47.90	112	<0.001
Right Arm FAT [%]	33.18	5.56	32.50	26.10	57.80	71	29.97	4.76	29.25	17.60	47.20	112	<0.001
Left Arm FAT [%]	33.69	5.64	33.30	26.00	53.70	71	30.76	5.31	29.95	15.60	51.40	112	<0.001
Trunk FAT [%]	15.95	6.47	14.90	3.00	41.70	71	14.59	5.55	13.00	3.00	34.90	112	<0.001
The content of adipose tissue in % (FAT %)	22.05	5.29	20.50	15.20	42.80	71	20.03	5.53	18.55	6.70	40.70	112	0.016
Lean tissue in kg (FFM kg)	77.94	5.31	79.44	57.14	84.62	71	79.97	5.55	81.27	59.22	93.21	112	0.015
Total water content in % (TBW %)	57.06	3.89	58.06	41.82	61.99	71	58.54	4.06	59.56	43.38	68.33	112	0.015
Muscle tissue in kg (PMM kg)	73.82	5.01	75.40	54.17	80.09	71	75.49	5.18	76.83	55.97	87.78	112	0.033

p - t-test for independent groups (taking into account the correction resulting from the homogeneity of variance (homogeneity was calculated using Levene's test)

Supplementary Table 3. Differences between girls and boys in body posture and body composition parameters after puberty.

Variables in puberty	Girls						Boys						p
	×	SD	Me	Min.	Max.	N	×	SD	Me	Min.	Maks.	N	
**Body posture**													
Total length [mm]	450.22	32.18	454.00	358.00	522.00	159	476.97	38.38	480.50	377.00	561.00	122	<0.001
Thoracic Length [mm]	318.78	27.47	318.00	241.00	391.00	159	335.89	29.22	335.50	268.00	404.00	122	<0.001
Lumbar Length [mm]	96.84	15.22	97.00	51.00	133.00	159	103.96	16.09	102.00	54.00	143.00	122	<0.001
Pelvic torsion [degree]	3.36	3.10	2.70	0.00	21.00	159	3.71	3.55	2.90	0.00	18.80	122	0.377
Pelvic obliquity [degree]	1.92	1.68	1.50	0.00	8.50	159	1.94	1.59	1.55	0.00	8.30	122	0.942
Pelvic/shoulder obliquity [degree]	2.35	1.83	1.80	0.10	9.20	159	4.12	14.89	2.40	0.00	165.50	122	0.139
Scapula distance RIGHT [mm]	44.77	12.43	44.00	10.00	88.00	159	55.93	13.63	56.50	26.00	91.00	122	<0.001
Scapula distance LEFT [mm]	43.57	12.73	44.00	15.00	89.00	159	53.66	13.12	54.00	25.00	83.00	122	<0.001
Scapula distance difference [mm]	6.67	5.38	5.00	0.00	27.00	159	6.81	5.91	5.00	0.00	28.00	122	0.838
Pelvic height difference RIGHT [mm]	4.74	7.04	1.00	0.00	29.90	158	4.98	7.53	0.40	0.00	37.40	122	0.780
Pelvic height difference LEFT [mm]	3.54	6.05	0.00	0.00	29.20	159	3.93	6.13	0.00	0.00	32.80	122	0.588
Shoulder height difference RIGHT [mm]	7.47	6.07	5.70	0.10	25.40	54	7.48	5.42	6.80	1.30	26.10	34	0.995
Shoulder height difference LEFT [mm]	11.06	7.82	9.75	0.10	41.80	104	11.75	8.84	10.65	0.50	59.80	88	0.567
Lateral inclination LEFT [degree]	0.62	0.90	0.10	0.00	5.10	158	0.79	1.06	0.30	0.00	6.40	122	0.154
Lateral inclination RIGHT [degree]	0.60	0.91	0.00	0.00	4.50	159	0.54	0.89	0.00	0.00	3.60	122	0.594
**Body composition**													
Right Leg FAT [%]	31.19	4.74	30.90	22.40	48.40	159	21.45	6.11	19.85	11.20	44.10	122	<0.001
Left Leg FAT [%]	31.46	4.77	31.10	23.10	49.60	159	22.04	6.09	20.15	11.90	43.40	122	<0.001
Right Arm FAT [%]	32.92	4.87	32.70	16.40	55.30	159	25.44	5.60	24.70	14.60	45.40	122	<0.001
Left Arm FAT [%]	35.74	5.99	35.70	23.10	61.10	159	26.66	6.23	25.35	17.20	52.00	122	<0.001
Trunk FAT [%]	18.86	5.99	18.30	6.40	42.40	159	14.93	6.26	13.20	5.60	40.50	122	<0.001
The content of adipose tissue in % (FAT %)	24.82	5.42	24.40	14.90	46.90	159	18.63	5.91	16.80	10.50	41.00	122	<0.001
Lean tissue in kg (FFM kg)	75.18	5.42	75.58	53.08	84.97	159	81.23	6.22	83.18	59.05	89.35	122	<0.001
Total water content in % (TBW %)	55.03	3.97	55.33	38.81	62.27	159	59.46	4.55	60.88	43.27	65.29	122	<0.001
Muscle tissue in kg (PMM kg)	71.32	5.12	71.72	50.41	80.67	159	77.08	5.88	78.98	56.03	84.54	122	<0.001

p - t-test for independent groups (taking into account the correction resulting from the homogeneity of variance (homogeneity was calculated using Levene's test)

Supplementary Table 4. Differences in girl's body posture and body composition parameters before and after puberty.

Variables	Girls before puberty						Girls after puberty						p
	×	SD	Me	Min.	Max.	N	×	SD	Me	Min.	Maks.	N	
**Body posture**													
Total length [mm]	374.27	28.88	371.00	314.00	457.00	71	450.22	32.18	454.00	358.00	522.00	159	<0.001
Thoracic Length [mm]	273.59	23.23	272.00	176.00	332.00	71	318.78	27.47	318.00	241.00	391.00	159	<0.001
Lumbar Length [mm]	73.28	15.59	74.00	34.00	105.00	71	96.84	15.22	97.00	51.00	133.00	159	<0.001
Pelvic torsion [degree]	4.53	3.77	3.50	0.40	17.90	71	3.36	3.10	2.70	0.00	21.00	159	0.015
Pelvic obliquity [degree]	2.30	2.12	1.70	0.10	8.10	71	1.92	1.68	1.50	0.00	8.50	159	0.188
Pelvic/shoulder obliquity [degree]	2.45	1.90	2.00	0.00	8.90	71	2.35	1.83	1.80	0.10	9.20	159	0.690
Scapula distance RIGHT [mm]	38.83	12.92	40.00	10.00	69.00	71	44.77	12.43	44.00	10.00	88.00	159	0.001
Scapula distance LEFT [mm]	50.01	78.11	40.00	14.00	688.00	71	43.57	12.73	44.00	15.00	89.00	159	0.492
Scapula distance difference [mm]	7.54	6.73	6.00	0.00	32.00	71	6.67	5.38	5.00	0.00	27.00	159	0.301
Pelvic height difference RIGHT [mm]	1.87	3.18	0.00	0.00	12.10	71	4.74	7.04	1.00	0.00	29.90	158	<0.001
Pelvic height difference LEFT [mm]	5.61	7.33	2.20	0.00	24.10	71	3.54	6.05	0.00	0.00	29.20	159	0.039
Shoulder height difference RIGHT [mm]	3.94	3.23	2.90	0.20	14.90	29	7.47	6.07	5.70	0.10	25.40	54	0.001
Shoulder height difference LEFT [mm]	13.72	8.37	14.00	0.70	32.80	42	11.06	7.82	9.75	0.10	41.80	104	0.070
Lateral inclination LEFT [degree]	0.98	1.16	0.50	0.00	4.90	71	0.62	0.90	0.10	0.00	5.10	158	0.025
Lateral inclination RIGHT [degree]	0.31	0.66	0.00	0.00	2.90	71	0.60	0.91	0.00	0.00	4.50	159	0.008
**Body composition**													
Right Leg FAT [%]	29.99	5.55	29.60	8.50	48.30	71	31.19	4.74	30.90	22.40	48.40	159	0.092
Left Leg FAT [%]	30.64	4.90	29.80	23.80	50.30	71	31.46	4.77	31.10	23.10	49.60	159	0.234
Right Arm FAT [%]	33.18	5.56	32.50	26.10	57.80	71	32.92	4.87	32.70	16.40	55.30	159	0.726
Left Arm FAT [%]	33.69	5.64	33.30	26.00	53.70	71	35.74	5.99	35.70	23.10	61.10	159	0.016
Trunk FAT [%]	15.95	6.47	14.90	3.00	41.70	71	18.86	5.99	18.30	6.40	42.40	159	0.001
The content of adipose tissue in % (FAT %)	22.05	5.29	20.50	15.20	42.80	71	24.82	5.42	24.40	14.90	46.90	159	<0.001
Lean tissue in kg (FFM kg)	77.94	5.31	79.44	57.14	84.62	71	75.18	5.42	75.58	53.08	84.97	159	<0.001
Total water content in % (TBW %)	57.06	3.89	58.06	41.82	61.99	71	55.03	3.97	55.33	38.81	62.27	159	<0.001
Muscle tissue in kg (PMM kg)	73.82	5.01	75.40	54.17	80.09	71	71.32	5.12	71.72	50.41	80.67	159	<0.001

p - t-test for independent groups (taking into account the correction resulting from the homogeneity of variance (homogeneity was calculated using Levene's test)

Supplementary Table 5. Differences in boy's body posture and body composition parameters before and after puberty.

Variables	Boys before puberty						Boys after puberty						p
	×	SD	Me	Min.	Max.	N	×	SD	Me	Min.	Maks.	N	
**Body posture**													
Total length [mm]	387.04	32.96	385.00	308.00	486.00	112	476.97	38.38	480.50	377.00	561.00	122	<0.001
Thoracic Length [mm]	283.65	25.38	282.50	217.00	392.00	112	335.89	29.22	335.50	268.00	404.00	122	<0.001
Lumbar Length [mm]	76.11	15.99	74.00	23.00	110.00	112	103.96	16.09	102.00	54.00	143.00	122	<0.001
Pelvic torsion [degree]	3.47	3.47	2.65	0.00	29.40	112	3.71	3.55	2.90	0.00	18.80	122	0.592
Pelvic obliquity [degree]	2.45	1.81	2.30	0.00	7.70	112	1.94	1.59	1.55	0.00	8.30	122	0.022
Pelvic/shoulder obliquity [degree]	2.93	2.21	2.60	0.00	10.40	112	4.12	14.89	2.40	0.00	165.50	122	0.403
Scapula distance RIGHT [mm]	42.32	13.10	41.50	11.00	84.00	112	55.93	13.63	56.50	26.00	91.00	122	<0.001
Scapula distance LEFT [mm]	42.96	11.77	42.00	15.00	78.00	112	53.66	13.12	54.00	25.00	83.00	122	<0.001
Scapula distance difference [mm]	7.58	7.01	6.00	0.00	41.00	112	6.81	5.91	5.00	0.00	28.00	122	0.364
Pelvic height difference RIGHT [mm]	4.92	6.36	0.30	0.00	23.20	112	4.98	7.53	0.40	0.00	37.40	122	0.950
Pelvic height difference LEFT [mm]	4.04	6.65	0.00	0.00	32.40	112	3.93	6.13	0.00	0.00	32.80	122	0.901
Shoulder height difference RIGHT [mm]	6.08	4.34	5.40	0.10	19.30	33	7.48	5.42	6.80	1.30	26.10	34	0.251
Shoulder height difference LEFT [mm]	10.97	8.87	8.40	0.10	39.90	79	11.75	8.84	10.65	0.50	59.80	88	0.571
Lateral inclination LEFT [degree]	0.86	1.20	0.10	0.00	6.20	112	0.79	1.06	0.30	0.00	6.40	122	0.638
Lateral inclination RIGHT [degree]	0.60	0.92	0.00	0.00	4.40	112	0.54	0.89	0.00	0.00	3.60	122	0.644
**Body composition**													
Right Leg FAT [%]	26.23	5.99	25.40	5.90	47.80	112	21.45	6.11	19.85	11.20	44.10	122	0.063
Left Leg FAT [%]	26.71	6.05	25.95	6.40	47.90	112	22.04	6.09	20.15	11.90	43.40	122	0.103
Right Arm FAT [%]	29.97	4.76	29.25	17.60	47.20	112	25.44	5.60	24.70	14.60	45.40	122	0.105
Left Arm FAT [%]	30.76	5.31	29.95	15.60	51.40	112	26.66	6.23	25.35	17.20	52.00	122	0.030
Trunk FAT [%]	14.59	5.55	13.00	3.00	34.90	112	14.93	6.26	13.20	5.60	40.50	122	0.657
The content of adipose tissue in % (FAT %)	20.03	5.53	18.55	6.70	40.70	112	18.63	5.91	16.80	10.50	41.00	122	<0.001
Lean tissue in kg (FFM kg)	79.97	5.55	81.27	59.22	93.21	112	81.23	6.22	83.18	59.05	89.35	122	<0.001
Total water content in % (TBW %)	58.54	4.06	59.56	43.38	68.33	112	59.46	4.55	60.88	43.27	65.29	122	<0.001
Muscle tissue in kg (PMM kg)	75.49	5.18	76.83	55.97	87.78	112	77.08	5.88	78.98	56.03	84.54	122	<0.001

p - t-test for independent groups (taking into account the correction resulting from the homogeneity of variance (homogeneity was calculated using Levene's test)

Supplementary Table 6. Differences in body posture and body composition parameters before and after puberty in all children.

Variables	All children before puberty						All children after puberty						*p*
	×	SD	Me	Min.	Max.	*N*	×	SD	Me	Min.	Maks.	*N*	
**Body posture**													
Total length [mm]	382.09	31.97	380.00	308.00	486.00	183	461.83	37.38	461.00	358.00	561.00	281	<0.001
Thoracic Length [mm]	279.75	24.99	278.00	176.00	392.00	183	326.21	29.45	326.00	241.00	404.00	281	<0.001
Lumbar Length [mm]	75.01	15.86	74.00	23.00	110.00	183	99.93	15.97	100.00	51.00	143.00	281	<0.001
Pelvic torsion [degree]	3.88	3.62	3.00	0.00	29.40	183	3.51	3.30	2.80	0.00	21.00	281	0.264
Pelvic obliquity [degree]	2.39	1.93	2.00	0.00	8.10	183	1.93	1.64	1.50	0.00	8.50	281	0.008
Pelvic/shoulder obliquity [degree]	2.74	2.10	2.30	0.00	10.40	183	3.11	9.92	2.00	0.00	165.50	281	0.617
Scapula distance RIGHT [mm]	40.97	13.11	41.00	10.00	84.00	183	49.62	14.08	50.00	10.00	91.00	281	<0.001
Scapula distance LEFT [mm]	45.70	49.42	41.00	14.00	688.00	183	47.95	13.82	48.00	15.00	89.00	281	0.470
Scapula distance difference [mm]	7.56	6.89	6.00	0.00	41.00	183	6.73	5.61	5.00	0.00	28.00	281	0.156
Pelvic height difference RIGHT [mm]	3.74	5.55	0.00	0.00	23.20	183	4.84	7.24	0.80	0.00	37.40	280	0.065
Pelvic height difference LEFT [mm]	4.65	6.95	1.00	0.00	32.40	183	3.71	6.08	0.00	0.00	32.80	281	0.136
Shoulder height difference RIGHT [mm]	5.08	3.98	4.15	0.10	19.30	62	7.47	5.80	6.50	0.10	26.10	88	0.003
Shoulder height difference LEFT [mm]	11.93	8.76	9.10	0.10	39.90	121	11.38	8.29	10.30	0.10	59.80	192	0.579
Lateral inclination LEFT [degree]	0.91	1.18	0.30	0.00	6.20	183	0.70	0.98	0.20	0.00	6.40	280	0.048
Lateral inclination RIGHT [degree]	0.49	0.84	0.00	0.00	4.40	183	0.57	0.90	0.00	0.00	4.50	281	0.288
**Body composition**													
Right Leg FAT [%]	27.69	6.09	26.90	5.90	48.30	183	26.96	7.23	27.80	11.20	48.40	281	0.246
Left Leg FAT [%]	28.23	5.93	27.30	6.40	50.30	183	27.37	7.12	27.90	11.90	49.60	281	0.157
Right Arm FAT [%]	31.21	5.30	30.10	17.60	57.80	183	29.67	6.38	30.30	14.60	55.30	281	0.005
Left Arm FAT [%]	31.89	5.61	30.70	15.60	53.70	183	31.79	7.57	31.80	17.20	61.10	281	0.870
Trunk FAT [%]	15.11	5.95	13.60	3.00	41.70	183	17.16	6.41	16.40	5.60	42.40	281	<0.001
The content of adipose tissue in % (FAT %)	20.81	5.52	19.60	6.70	42.80	183	22.13	6.42	21.70	10.50	46.90	281	0.019
Lean tissue in kg (FFM kg)	79.18	5.53	80.41	57.14	93.21	183	77.81	6.50	78.36	53.08	89.35	281	0.015
Total water content in % (TBW %)	57.97	4.05	58.80	41.82	68.33	183	56.96	4.76	57.30	38.81	65.29	281	0.015
Muscle tissue in kg (PMM kg)	74.85	5.16	75.97	54.17	87.78	183	73.82	6.16	74.16	50.41	84.54	281	0.054

p - t-test for independent groups (taking into account the correction resulting from the homogeneity of variance (homogeneity was calculated using Levene's test)
